# Kathu Townlands: A High Density Earlier Stone Age Locality in the Interior of South Africa

**DOI:** 10.1371/journal.pone.0103436

**Published:** 2014-07-24

**Authors:** Steven J. H. Walker, Vasa Lukich, Michael Chazan

**Affiliations:** 1 Department of Archaeology, University of Cape Town, Rondebosch, South Africa; 2 Department of Earth Sciences, University of Toronto, Toronto, Ontario, Canada; 3 Department of Anthropology, University of Toronto, Toronto, Ontario, Canada; University of Oxford, United Kingdom

## Abstract

Kathu Townlands is a high density Earlier Stone Age locality in the Northern Cape Province, South Africa. Here we present the first detailed information on this locality based on analysis of a sample of lithic material from excavations by P. Beaumont and field observations made in the course of fieldwork in 2013. The results confirm the remarkably high artefact density at Kathu Townlands and do not provide evidence consistent with high energy transport as a mechanism of site formation, suggesting that Kathu Townlands was the site of intensive exploitation of highly siliceous outcroppings of banded iron formation. The results presented here provide a first step towards understanding this complex locality and point to the need for further research and the importance of preserving this locality in the face of intensive and rapid development.

## Introduction

**Figure 1:**
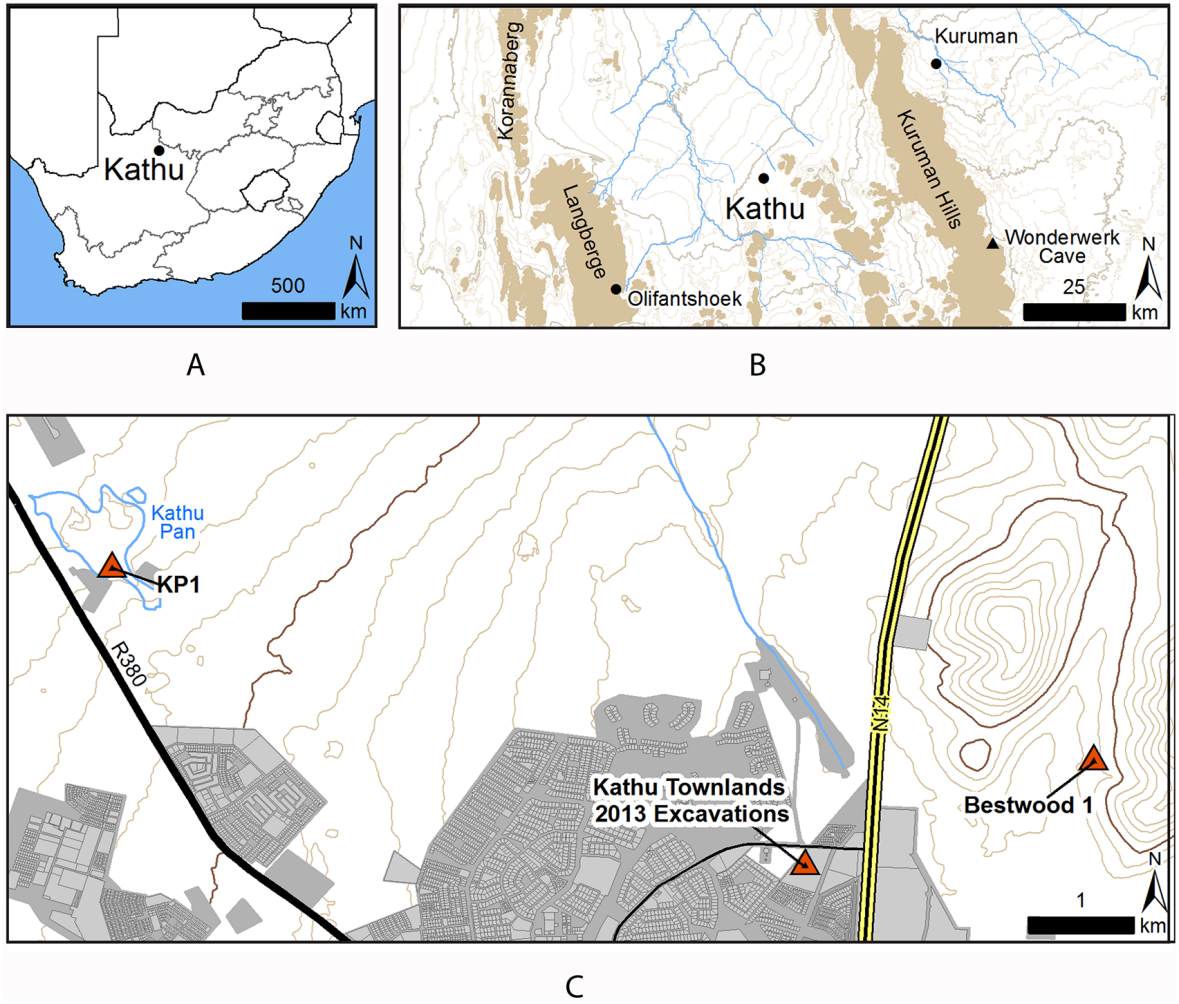
Location of Kathu Townlands. A. Location map within South Africa. B. Location Map with relation to the regional topography and Wonderwerk Cave. C. Topographic context of sites of the Kathu Complex discussed in this article. Grey shading indicates developed areas and areas undergoing development. Note that the boundaries of Kathu Pan are approximate and do not indicate the limit of areas of archaeological potential.

**Figure 2:**
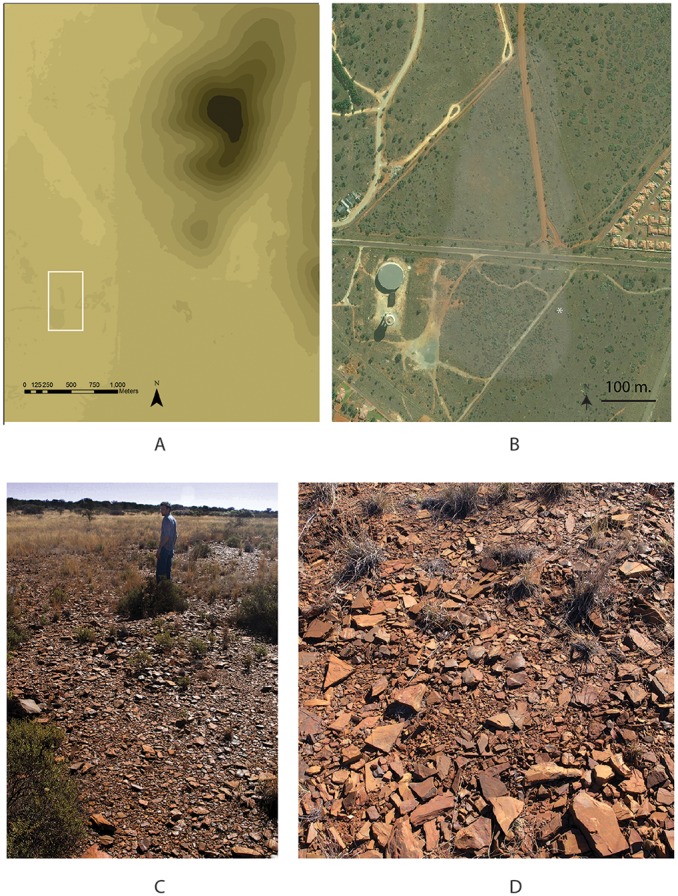
Views of Kathu Townlads. A. Digital Elevation Model (DEM) showing the topographic context of Kathu Townlands. Square shows the approximate area shown in Figure 2b. (DEM courtesy of Stephen Wessels, The Zimani Project). B. Aerial photo of Kathu Townlands. White shading indicates approximate limits of the declared locality. Asterisk indicates location of 2013 excavations. Image dated 5/27/2011 predates recent development south of Frikkie Meyer St. Image source Terraserver. C. View of Towlands site north of Frikkie Meyer St. D. Detail showing scatter of artefacts on the surface of the site north of Frikkie Meyer St.

Early to Middle Pleistocene localities that incorporate extremely high numbers of lithic artefacts present a challenge to our understanding of early hominin behaviour. In some cases, such as Ma’ayan Baruch, Israel [1], the deposits are dominated by very large numbers of bifaces while other localities show characteristics consistent with quarrying or primary production [2], [3], [4], [5]. There are also localities with a more ambiguous character such as the site of Canteen Kopje, South Africa [6], [7] which includes high densities of both production debris and finished artefacts. Both the quarry sites and sites dominated by finished artefacts provide evidence of stone tool transport but these sites also raise questions about group size and organization of activity among early hominin groups. Here we present the first detailed description of a site located in the town of Kathu, Northern Cape Province, South Africa that shows high intensity of lithic production during the Earlier Stone Age (ESA). Kathu Townlands is a site situated between the Kuruman Hills to the east and the Langberge mountains to the west on a low hill and is covered with a dense surface of lithics interspersed with exposures of bedrock, calcrete, and sand (Figure 1–2).

Dense and broadly distributed archaeological deposits pose methodological and management challenges. The town of Kathu is rapidly expanding and this development is directly threatening Kathu Townlands (Figure 1c). The site was designated a Grade 1 National Heritage site in 2013 however the threat to deposits beyond the declared area remain acute.

### Geological Setting

The bedrock lithology is Precambrian, with exposures of banded iron formation (BIF), which belong to the Kuruman Formation within the late Archean to earliest Paleoproterozoic Transvaal Supergroup [8], [9]. There is a wide degree of variability within the BIF in the Kuruman Formation both in terms of the scale of banding and the percentage of chert (SiO_2_) relative to iron-rich minerals. The outcroppings at Kathu Townlands (sometimes designated as jaspellite) are dominated by chert and show no fine-scale banding. As a result the structure of these rocks is ideal for stone tool manufacture and it is likely that the availability of high quality raw material is a major reason for repeated exploitation of the resource and the high density of stone tool and knapping debris at Kathu Townlands. Circular white to grey fossil traces are characteristic of the raw material found at Kathu Townlands. Outcroppings of raw material with similar fossil traces have not been identified in the surrounding region.

The Kuruman Hills are today drained by a series of ephemeral streams that flow northwest (Figure 1). None of these streams pass through the research area, the closest drainage is the Vermulsleegte, to the north of the site. There is evidence for far more substantial drainage systems at some point in the geological past in the area around Kathu. At the Bestwood site the archaeological horizon is underlain by at least ten meters of river gravels and similar deposits are known from other localities in the region, although no such deposits are known at Kathu Townlands.

Calcretes are a common feature in the area around Kathu and figure significantly in the deposits in the vicinity of Kathu Townlands. Calcretes develop in arid or semi-arid environments as the result of lateral and vertical movement of carbonates in solution but the diagenetic process causing the development of these deposits can vary depending on local conditions [10]. At the Mamatwan Mine near Hotazel, a calcrete horizon approximately 2 meters thick produced optically stimulated luminescence ages of 113,000 and 108,000 years ago [11]. Without detailed analysis it is not possible to determine the age of the calcrete deposits at Kathu Townlands.

A sand sheet, derived from the Kalahari, is found across the surface at Kathu Townlands and comprises the matrix within which most of the artefacts are found. As with calcretes, there were likely multiple cycles of sand accumulation in the Kathu area. Research at Wonderwerk Cave has demonstrated that Kalahari sands were blowing into this region by 2 million years ago [12] and it is likely that the sands at the base of Kathu Pan 1 are of such an early age. At the Bestwood 1 site, sands overlie the archaeological horizon and are thus of a younger age. At the Mamatwan Mine the Kalahari sands produced optically stimulated luminescence ages ranging from 62–44,000 years ago.

### Archaeological Setting

Kathu Townlands is a component of a grouping of ESA localities designated as the Kathu Complex. This complex also includes the excavated sites of Kathu Pan1 (KP1) and Bestwood 1 (BW 1, Figure 1c). At Kathu Pan, evidence of early hominin occupation has been observed at multiple locations within the pan, but ESA deposits have only been excavated at KP 1 [13]. Stratum 4a at KP1 is dated by a combination of OSL and ESR/U-series to ca. 500 k BP [13]. The lithic assemblage from St. 4a is characterized by a prepared core technology that produced both blades and points, and has been attributed to the Fauresmith industry [14], [15]. The lithic assemblage of the underlying St. 4b at Kathu Pan 1 is characterized by well-made handaxes. At BW 1, located to the east of Kathu Townlands in a valley between two small hills, mining of sand has revealed a horizon at the interface of gravels and the overlying sands that contains abundant lithic artefacts [16]. These are characterized by bifaces, blades, and prepared cores and are dispersed over a very large area. Excavation at BW1 in 2012 exposed a surface of 36 m^2^ with over 1000 piece plotted artefacts recovered. All artefacts were observed lying flat on at the interface between the sands and gravels. The extremely fresh condition of the lithic artefacts at Bestwood 1 argues against this accumulation being a palimpsest deposited over a long period, or as the result of deflation. The possibility that the concentration of artefacts in a single horizon is the result of bioturbation also seems unlikely [17]. The excavated area is apparently representative of an extensive occupation covering several hectares.

Kathu Townlands is located approximate 56 km. west of Wonderwerk Cave where a sequence of ESA occupation has produced early evidence of fire [18], [19]. At Wonderwerk the ESA occupation is characterized by low artefact density in marked contrast to Kathu Townlands.

### Previous Work at Kathu Townlands

The archaeological deposit at Kathu Townlands was brought to the attention of archaeologists in 1980 by Naas Viljoen, the manager of the property [20], [21]. The site was first described in a permit report submitted to the National Monuments Council outlining the results of a large scale survey for archaeological resources in the region [22]. Shortly afterwards, the outcropping of ironstone (and the tremendous amount of artefacts) was used as a source of road gravel. Mr Viljoen notified Mr Beaumont (who was excavating at KP1 at the time) that he had observed workmen using gravel that was composed primarily of artefacts to repair roads [20]. Excavations at the site were conducted by Beaumont in 1982, and then again in 1990 [20], [21]. Unfortunately, the precise location of these excavations was not reported.

There has also been a general lack of clarity regarding of the extent of the deposit. The initial reports identified an area north of Frikkie Meyer Street. Since at least 1990 the deposit has been known to exist south of this road. The total extent of the deposit, particularly beneath the surface sands, remains to be determined.

## Methods

Field work was conducted under the South African Heritage Resources Agency permit #577 (CaseID: 2992). McGregor Museum, Kimberley is the repository for all collections described here as well as our data (inventory number 6512). Analysis can be arranged by permission from the McGregor Museum.

### Lithic Analysis

The complete collection from one ten centimetre deep spit from Beaumont’s 1 m by 1 m excavation (Square K6 20–30 cm.) was chosen for complete analysis to provide a baseline for future studies. The McGregor Museum, Kimberley is the repository for all collections (inventory number 6512) and future analysis of these collections can be arranged by permission from the McGregor Museum. A sample of 20 handaxes from Beaumont’s surface collections was added to allow for analysis of handaxe morphology. The size of the assemblage from this context is representative of all excavated contexts below covering sands at Kathu Townlands. Measurements taken on flakes were limited to maximum dimension, degree of fragmentation (proximal, distal, medial, complete), condition (fresh, edge damage, abraded), raw material, weight, and flake type (primary: cortex >80%, secondary: cortex <80%, non-cortical). For cores, measurements taken were limited to description and weight. For bifaces, B1 (breadth 1/5 from the tip) and B2 (breadth 1/5 from the base) were measured along with length, maximum thickness, and weight. A subsample was selected for photography. The sample from this single spit includes 1283 flakes, 10 cores, and 6 bifaces (See Table 1).

**Table 1:** Breakdown of the sample of flakes and flake fragments from the Beaumont excavation (n = 1283).

Fragment		
	n	%
Complete	714	55.65%
Proximal	223	17.38%
Distal	191	14.89%
Medial	155	12.08%
**Condition**		
	**n**	**%**
Fresh	297	23.15%
Edge damage	879	68.51%
Abraded	107	8.34%
**Type**		
	**n**	**%**
Primary	72	5.61%
Secondary	222	17.30%
Non-cortical	989	77.08%

### 2013 Excavations

**Figure 3:**
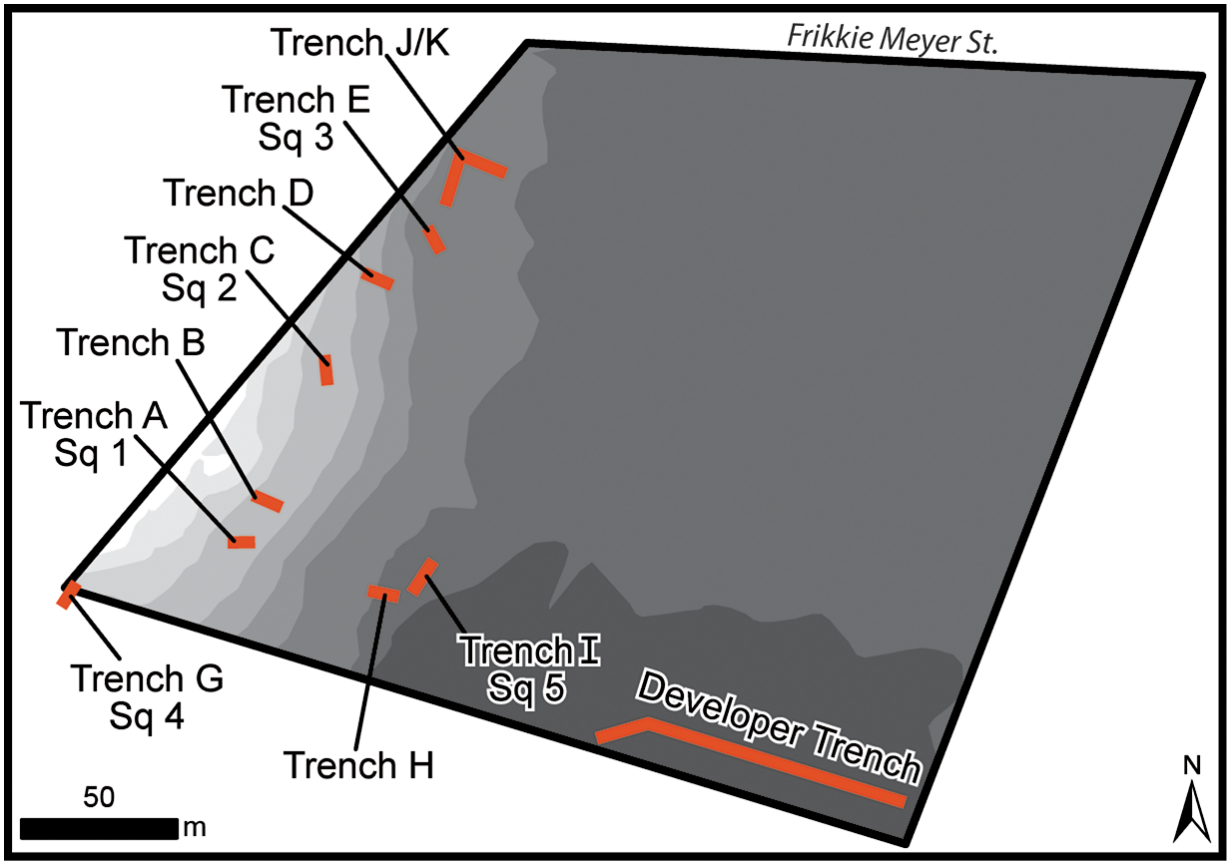
Plan of trenches and excavation units from the 2013 excavations. The topographic map was produced by a surveyor for the mall construction before any surface modifications were made to the project area. Note that the topography dips towards the west showing that the site occurs along a N-S ridge. This ridge is visible in the DEM in Figure 2a.

In August 2013 excavations were undertaken as part of a data recovery project to mitigate the destruction being caused by a mall being built on a small portion of the known deposit [23] (see Figure 2b, 3). The primary objective of the project was to recover samples from a controlled stratigraphic context distributed across the area slated for destruction in the limited time available. We aimed to obtain data useful for understanding both the horizontal and vertical distribution of the deposits over as wide an area as possible.

The methods employed combined mechanical trenching to obtain data regarding the geomorphology, followed by hand excavations to obtain archaeological samples (Figure 3). Horizontal control across the project was maintained with averaged GPS waypoints. Vertical associations were able to be correlated using a high resolution topographic map of the project area (20 cm intervals; Figure 3).

Under archaeological supervision, ten trenches were mechanically excavated across the property. The location of trenches was chosen with the intent of providing a cross section of the topography of the impacted area. Each of the ten trenches was cleaned to bedrock by hand and profiles were cleaned, measured and photographed. Additionally, a long trench dug by the developer was documented in an area that had been considered to be outside the site boundary.

From these ten trenches, five were selected for further archaeological excavation to provide a horizontally distributed sample from trenches that were archaeologically rich. The first three units were 100 cm×50 cm, however, due to the overwhelming amount of material being recovered, the last two were reduced to 50×50 cm (see Table 2). These units were dug in 10 cm levels as measured from modern ground surface (MGS). Vertical control was maintained utilizing a datum and line level. Each unit was dug to bedrock, all material was screened through a 3 mm sieve, and artefacts collected by level. All excavated material is curated in the McGregor Museum, Kimberley.

**Table 2:** Weight of lithic artefacts recovered in 2013 excavation by excavation unit broken down by arbitrary 10

	Trench A (*square 1)*	Trench C *(square 2)*	Trench E *(square 3)*	Trench G *(Square 4)*	Trench I (*Square 5)*
Opening Elevation	1232.9 m. asl 1233.1 m. asl 1232.1 m. asl 1233.3 m. asl 1231.9 m. asl				
Cm. Below Surface	Weight of artifacts (kg.)				
0–10	13.4		0.1	0.1	0.1
10–20	20.4	20.2*	0.1	0.2	0.2
20–30	17.2	17.4	0.6	4.4	0.2
30–40	21	17	0.6	15.2	0.1
40–50	27.2	11.4	3.6	8.2	4.8
50–60	14.8	12.6	18.4	4.4	9.4
60–70	14.8	10.4	4	7.4	7.2
70–80	22.8	15.2	7.6	3.6	0.8
80–90	14.4	4.2	5.8	5.2	0.6
90–100	22	BEDROCK	2.8	10.8	0.1
100–110	9.4		3.2	7.4	BEDROCK
110–120	13.4		0.1	2.4	
12– = 130	13.8		BEDROCK	0.1	
130–140	7			BEDROCK	
140–150	5.8				
150–160	12				
160–170	16.8				
170–180	15.8				
	BEDROCK				
Closing Elevation	1231.1	1232.2	1231.1	1232.0	1230.9
Area Excavated	100×50 cm.	100×50 cm.	100×50 cm.	50×50 cm.	50×50 cm.

*Square 2: Levels 1 & 2 were dug as a single spit.

## Results

### Lithic Analysis

**Figure 4:**
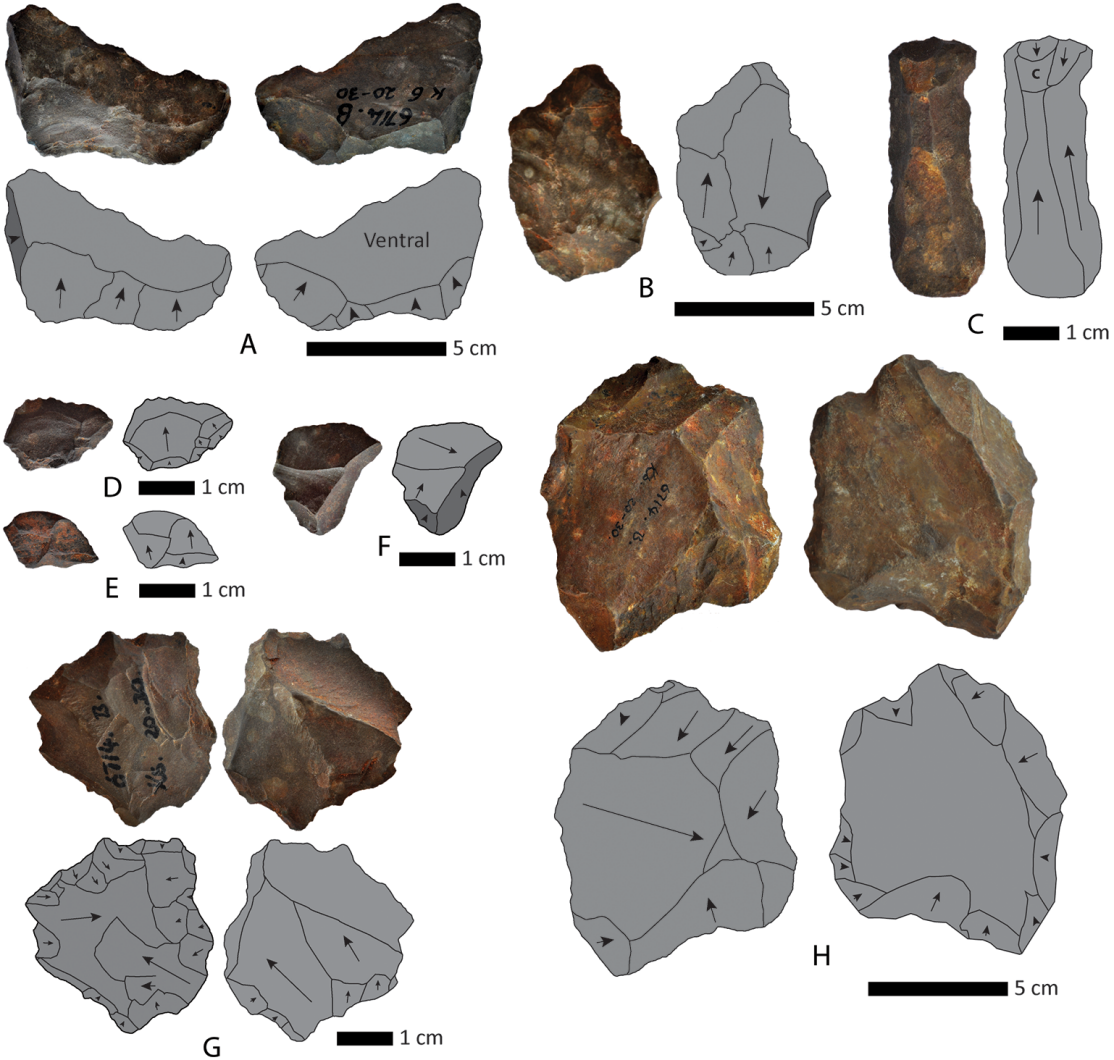
Flakes and cores from Kathu Townlands, Beaumont Excavation. A. Large flake off of the edge of the core consistent with biface shaping removal. B. Large flake with centripedal dorsal scars. C. Blade, note that there is some cortex (indicated by C in the sketch) and that scars are not parallel. D–F. Small flakes, note that F is off the edge of the core. G. Discoidal core with removals off both faces. Break on one edge (upper edge in right view). H. Discoidal core with one large flake removal. Note that on the right hand face the working is unclear and it is possible that this is a natural surface.

The breakdown of the analyzed lithic assemblage is presented in Table 1. The flakes include 714 (55.65%) complete pieces, 223 (17.38%) proximal fragments, 191 distal fragments (14.89%), and 155 medial fragments (12.08%) (Figure 4). Only 297 pieces are fresh (23.15%, sharp edges with little microfracture), 879 have edge damage (68.51%, considerable microfracture, crushing, and/or large scar damage along the edge), and 107 pieces are abraded (8.34%, smoothing due to abrasion, with or without edge damage). Maximum dimension for the large flakes has a mean of 4.6 cm (SD 2.28), and the mean for weight is 31.6 g (SD 55.9). The sample from the Beaumont excavation shows high fragmentation and a high degree of edge damage indicating significant mechanical damage. Flakes smaller than 2-cm. in maximum dimension are underrepresented, possibly due to the mesh size employed in sieving. However, there is no evidence for winnowing of flakes greater than 2 cm. as would be expected under high energy transport. Abrasion, although present, is not frequent.

**Figure 5:**
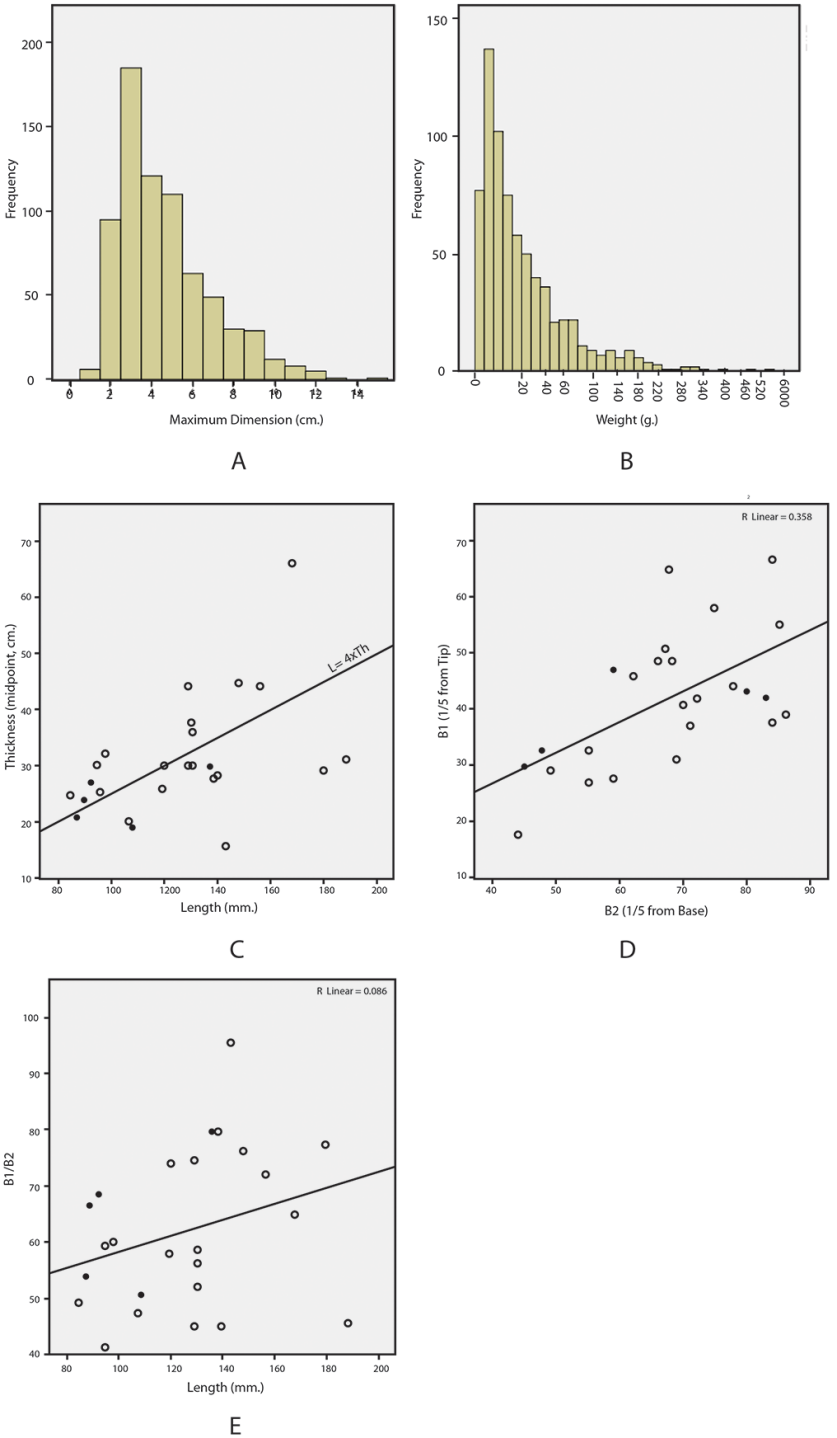
Metric attributes of flakes from Beaumont Excavation and bifaces from Beaumont Excavation and Surface collection. A. Frequency of maximum dimension for flakes. B. Frequency of weight for flakes. C–E. Metric attributes of bifaces (based on [25]). Beaumont excavation indicated by solid dot, surface collection by outline dot.

The representation of all size classes in the flake assemblage suggests that knapping took place on site and is consistent with the identification of the site as a quarry locality (Figure 5: a–b). However, the low frequency of primary flakes (n = 72, 5.61%) and secondary flakes (n = 222, 17.3%) and the high frequency of noncortical flakes (n = 989, 77.08%) does not match expectation for a quarry site where the initial stages of roughing out a block would take place.

Due to the frequency of edge damage, clearly intentional retouch is difficult to identify in the sample. Examples of clear retouch are rare (n = 10) consisting of side scrapers, retouched pieces, and one notch. Cores are extremely variable in morphology and include an amorphous core lacking any evident organization of flaking surfaces (n = 1) centripedal Levallois (n = 1), unidirectional (n = 1), cores on flakes (n = 2), and possible bifacial preforms (n = 2), and discoidal (n = 3), (Figure 4: g–h). Flakes that are typologically Levallois, and blades are found in the assemblage but are rare (Figure 4b–c). Biface thinning flakes are absent but many of the flakes could be derived from stages of biface manufacture (Figure 4a). The raw material for the entire flake assemblage is the local BIF.

**Figure 6:**
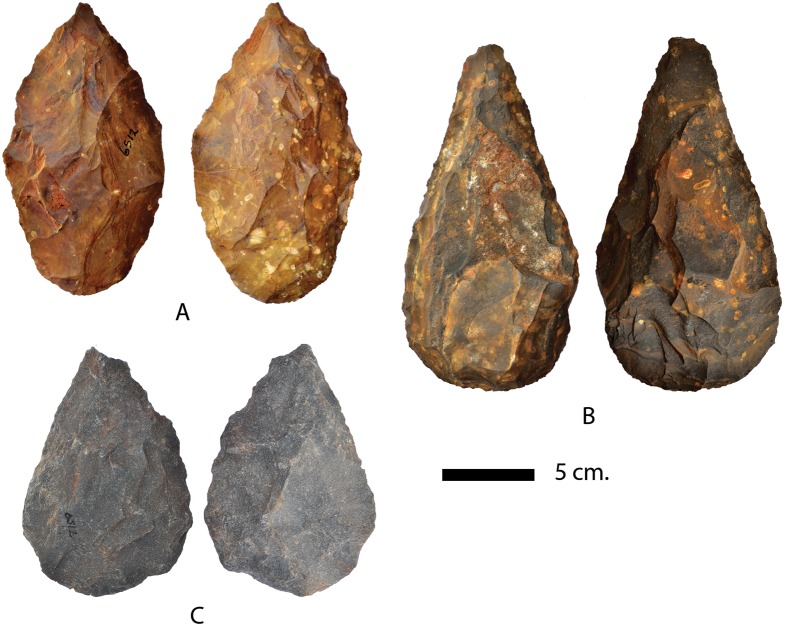
Handaxes from surface collection: A–B. Banded Ironstone. C. Quartzite.

The biface sample from the excavation unit is highly variable in shape (Figure 5: c–e, Figure 6). By comparison the selected sample from the surface collection conforms more regularly to the morphology of pointed handaxes. Handaxes show both large shaping scars and smaller removals along the edges. None of the handaxes show scars from arched invasive biface thinning flakes. Raw material for handaxes is heavily dominated by local BIF, however rare examples on quartzite are also found (Figure 6c).

### Excavation

**Figure 7:**
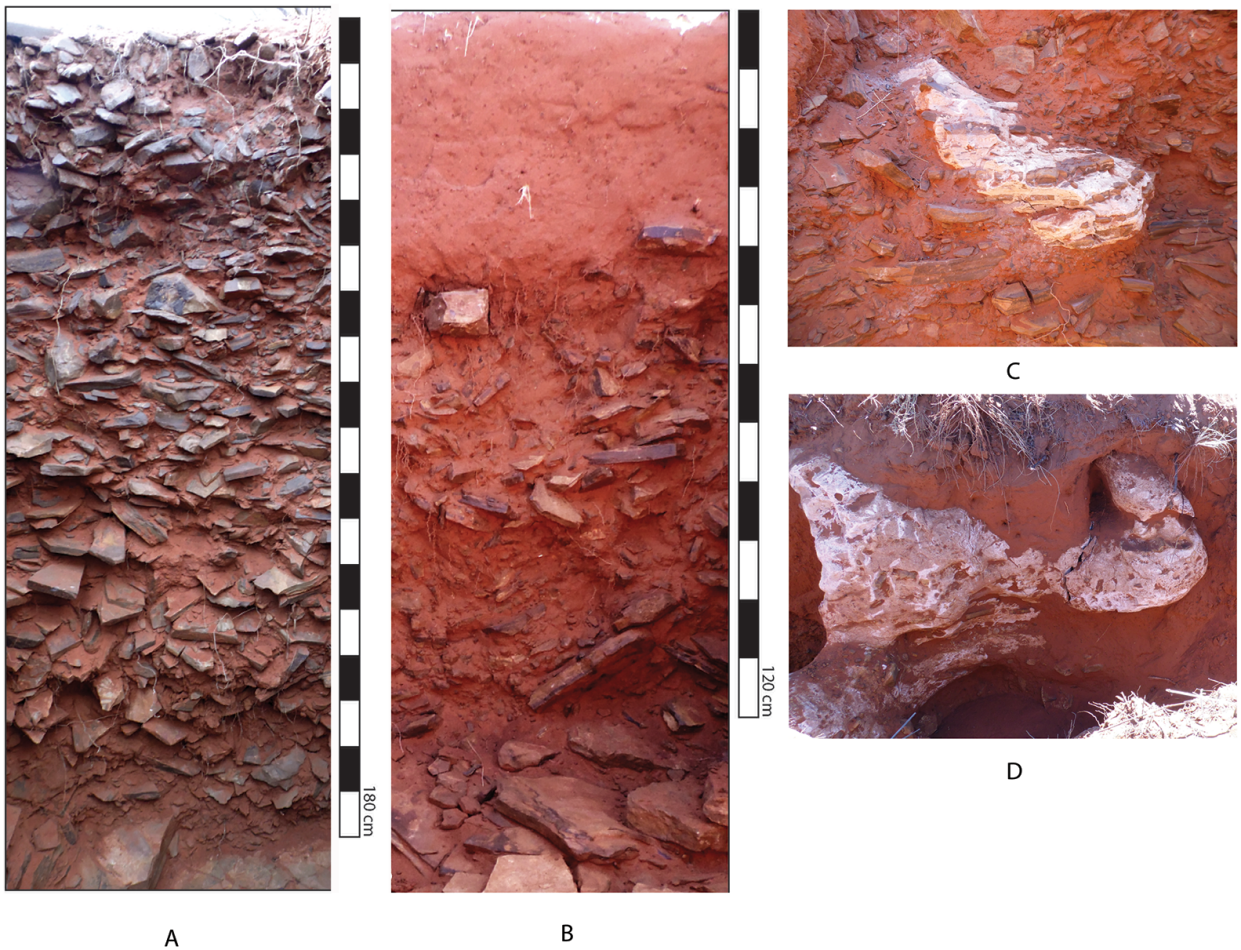
Profiles from 2013 excavation. A. Trench A: Square 1. Massive deposit of Banded Irontone rubble and artefacts overlying bedrock in a sandy matrix. Note lack of bedding or sorting. B. Trench I: Square 5. Shallow massive deposit of Banded Ironstone rubble and artefacts overlying bedrock with overlying deposits of sand. C. Trench E: Square 3. Discrete calcrete nodule that developed near the interface of the rubble/artefact deposit and underlying bedrock. Note parallel bedding of the Ironstone within the calcrete nodule. Approximate width of image 50 cm. D. Trench J/K. Discrete nodular calcrete developing in the sand and into the underlying Banded Ironstone rubble. Does not exhibit parallel Ironstone bedding found in (c). Approximate width of images 50 cm.

The excavated trenches exposed dense artefact deposits mixed with BIF rubble and sand reaching a maximum depth of 2.2 m in Trench B. A massive quantity of artefacts was recovered conforming to Beaumont’s observations. No stratigraphy was evident within the artefact horizon and artefacts and natural slabs appear randomly oriented within the profiles (Figure 7: a–b). The artefact horizon sits directly on bedrock in all units where bedrock was reached. There is thus an unconformity of over 2 billion years between the bedrock and the overlying rubble. In most units the archaeological horizon is overlain by sand mixed with very small (1–2 cm.), subangular pebbles. The contact between the archaeological horizon and the overlying sands is abrupt.

**Figure 8:**
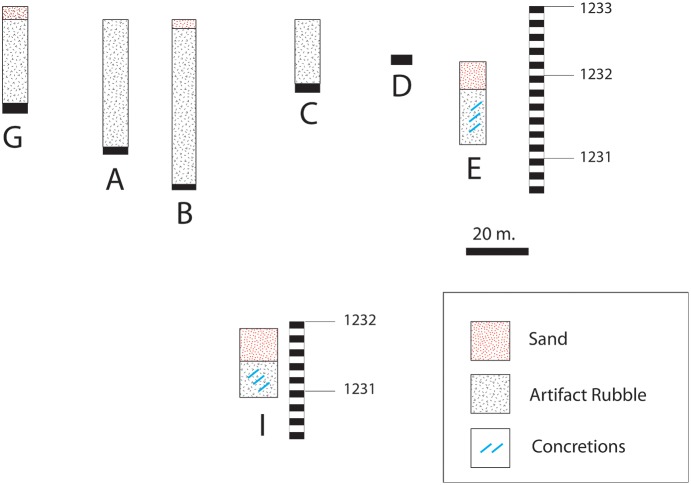
Composite profile along 2013 excavation trenches.

The bedrock topography does not conform to the modern land surface along the main NE-SW transect (Figure 8). Modern topography along this transect rises gently from NE to SW but the bedrock rises sharply between trenches E and D (ca. 1 m rise over 10 m). The bedrock topography then dips again into a channel centred on Trench B before rising again in Trenches A and G. The archaeological horizon appears to fill the irregularities in the bedrock topography of the channel. Moving to the SE, where the current topography drops rapidly, the archaeological horizon thins.

Calcium carbonate concretions (calcretes) develop within the archaeological horizon at lower elevations (Trenches E and I; Figure 7: c–d). In trenches J/K and the developer’s trench calcretes were prevalent although not consolidated into a continuous horizon as has been documented in other contexts in the region. The calcretes in these trenches develop within sands however it appears that aspects of the archaeological horizon are also incorporated into the calcretes.

**Figure 9:**
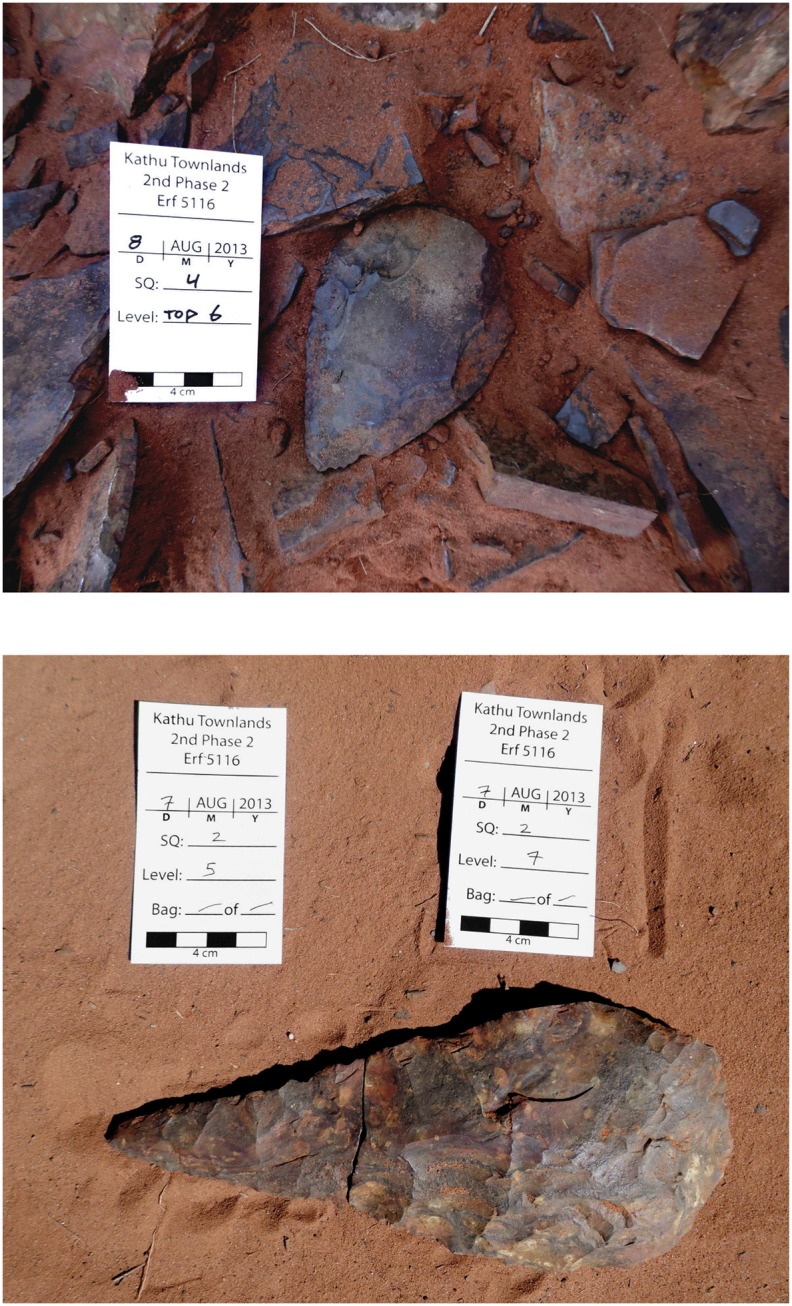
Artefacts from the 2013 excavation. A. Biface *in situ* in Square 4 (Trench G). B. Broken biface found in Square 2 (Trench C). Tip found in Level 5 (50–60 cm below surface), base found in Level 7 (70–80 cm. below surface).

Analysis of the archaeological assemblage recovered has not yet been carried out however a number of field observations provide insight into the formation of the archaeological horizon. All artefacts recovered are consistent with an Acheulean age and are similar to those found in Beaumont’s excavation. Included are a small number of well-made handaxes (Figure 9a). In Square 2 the pieces of a refitting broken handaxe were recovered; the tip from Level 5 and the base from Level 7 (Figure 9b). This observation suggests redeposition of the sediments that make up the archaeological horizon. During excavation of Square 1 a prevalence of larger artefacts was noticed in the upper levels (to a depth of ca. 70 cm), and an excess of smaller debitage in the lower levels. In Square 2 a large slab of bedrock was encountered within the artefact horizon, roughly 40 cm above actual bedrock.

## Discussion

The Kathu Townlands site is, as previously reported, a massive deposit of Acheulean artefacts likely the product of the exploitation of local BIF. Preliminary analysis of lithic material and fieldwork in a mitigation context provide some initial observations about site formation. The archaeological horizon is a dense rubble of artefacts and unworked BIF in a sand matrix. It is possible that the sands are derived from the overlying deposits rather than an initial component of the archaeological horizon. The archaeological horizon sits directly on the bedrock and appears to fill in irregularities in the bedrock topography, including a channel apparent in the NE-SW transect. Analogues to this situation can be found on the ironstone hills on the Bestwood farm about 1 km to the east where artefacts sit directly on the exposed bedrock.

Both lithic analysis and field observations failed to find compelling evidence of high energy transport. Polishing of artefacts is rare, clasts are angular, and there is little evidence of sorting. There is however considerable evidence of mechanical damage to artefacts (randomly organized microfracture on edges). This observation is supported by the recovery of a broken handaxe found in levels separated by 20 cm within Square 2 (Figure 9b).

As a working hypothesis, these observations suggest that at the time of occupation Kathu Townlands was a small exposed hill of chert-rich BIF. This locality was the site of ongoing intensive occupation and exploitation for stone tool manufacture. While one function of the site might have been as a quarry, rough-outs and primary flakes are rare, and there is a small component of finished tools (including rare handaxes made on non-local quartzite) suggesting that the site might have had a more diversified function. The artefact horizon was subject to mechanical forces and redeposition. The mechanisms responsible for these processes are currently unclear but high energy water transport does not appear to have played a role. The age of the covering sands and the development of calcretes remains to be explored, however it is possible that these are Late Pleistocene to Holocene in age as is the case for dated sands and other calcretes in the region [11].

Kathu Townlands represents a complex and massive archaeological context that requires further research. The occurrence of a low density Acheulean occupation at Wonderwerk Cave suggests significant differences in the intensity of hominin activity between the two flanks of the Kuruman Hills. It is likely that the density of archaeological remains within the Kathu Complex is related to local availability of water. It is intriguing to consider the possibility that the hominin occupation of Wonderwerk Cave during the Acheulean is the result of seasonal mobility of small groups of hominins dispersing from the core occupation area on the western flanks of the Kuruman Hills.

Given the rapid development of the town of Kathu, long term protection of the deposits at Townlands is imperative. However, simply preserving isolated artefact-dense patches of land will not preserve the information necessary to interpret the Kathu Complex. Preservation cannot be limited to the presence and absence of surface archaeological material. In Figure 2b the area of the site is intentionally indicated by shading rather than a distinct boundary. The concept of site boundaries that play such a critical role in heritage resource management is inherently problematic [24]. A broader landscape-based effort of subsurface testing including palaeo-landcape and palaeo-environmental reconstruction is essential to our understanding of this extraordinary record. Sources of this information must be protected along with archaeological remains. Together with the other components of the Kathu Complex, this site represents a high density of hominin occupation that presents a challenge to reconstructions of hominin adaptations during the Early-Middle Pleistocene. Hopefully, enough of this record will be preserved to allow for ongoing reconstruction and investigation of this important scientific resource.
